# Prevalence of *Fms‐Like Tyrosine Kinase 3 (FLT3)* Mutations in Patients With Acute Myeloid Leukaemia: A Systematic Literature Review and Meta‐Analysis

**DOI:** 10.1002/cam4.71205

**Published:** 2025-09-22

**Authors:** Juliana F. M. Lewis, Naval G. Daver, Noah Jamie Robinson, Bhavik J. Pandya, Bosny Pierre‐Louis, Sayma Monir, Jorge Sierra

**Affiliations:** ^1^ Astellas Pharma, Inc. Northbrook Illinois USA; ^2^ Department of Leukemia The University of Texas MD Anderson Cancer Center Houston Texas USA; ^3^ Astellas Pharma A.G. Wallisellen Switzerland; ^4^ Astellas Pharma Europe B.V. Leiden the Netherlands; ^5^ Department of Hematology Hospital Santa Creu i Sant Pau, IR‐Sant Pau, and José Carreras Leukemia Research Institute Barcelona Spain

**Keywords:** acute myeloid leukaemia, *fms‐like tyrosine kinase 3*, meta‐analyses, mutation, prevalence, systematic literature review

## Abstract

**Background:**

*Fms‐like tyrosine kinase 3* (*FLT3*) mutations are associated with poor prognosis in patients with acute myeloid leukaemia (AML).

**Aims:**

We conducted a systematic literature review and meta‐analyses of studies reporting *FLT3* mutation prevalence in patients with AML.

**Materials & Methods:**

We searched all publications through September 2022; the earliest publication we retrieved was published in 1997. Based on these publications, data from the studies were generated between 1985 and 2021. Prevalence was evaluated overall and by study type, geographic location of study, patient age, and gender.

**Results:**

Weighted mean (95% confidence interval) prevalence for *FLT3* internal tandem duplication (ITD) and *FLT3* tyrosine kinase domain (TKD) mutations were 20% (19%–22%) and 7% (6%–8%), respectively, with wide variability in individual study estimates (*FLT3*‐ITD: 5.1%–41.4%; *FLT3*‐TKD: 2.3%–12.0%). Weighted mean prevalence estimates for *FLT3‐*ITD and *FLT3‐*TKD mutations were higher in populations from interventional (*FLT3‐*ITD: 22%; *FLT3‐*TKD: 8%) than non‐interventional studies (*FLT3‐*ITD: 19%; *FLT3‐*TKD: 6%). Weighted mean *FLT3* mutation prevalence estimates were higher for Europe (*FLT3‐*ITD: 23%; *FLT3‐*TKD: 8%) and lower for Asia (*FLT3‐*ITD: 18%; *FLT3‐*TKD: 5%). Weighted mean prevalence of *FLT3*‐ITD mutations was higher in younger adults (aged 18–59 years; 23%) than paediatric (aged < 18 years; 12%) or older (aged ≥ 60 years; 18%) populations, and in females (22%) than males (18%).

**Discussion:**

This was the first study to comprehensively assess the reported prevalence of *FLT3* mutations worldwide among AML patients.

**Conclusion:**

We described the distribution of *FLT3* mutations; further work is needed to understand prevalence estimate heterogeneity.

## 1 Introduction

Acute myeloid leukaemia (AML) is a heterogeneous haematological malignancy that is characterised by uncontrolled proliferation of leukaemic stem cells [1, 2, 3]. In 2021, the global number of incident cases of AML was approximately 144,000, with over 130,000 AML‐related deaths in the same year [4]. This disease predominantly affects the elderly, with the highest number of disability‐adjusted life years and deaths associated with AML among patients aged ≥ 70 years [4].

*Fms‐like tyrosine kinase 3 (FLT3)* mutations, including internal tandem duplications (ITD) and point mutations in the tyrosine kinase domain (TKD), are among the most common genetic aberrations associated with AML [1, 2, 3]. *FLT3*‐ITD mutations are associated with particularly poor prognosis in patients with AML, including high relapse rates and low survival rates [2, 3]. The prognostic implications of *FLT3*‐TKD mutations in patients with AML are less clear and are thought to depend on the existence of co‐mutations or cytogenetic changes [1, 2, 3]. Patients with AML who have *FLT3* mutations may benefit from treatment with *FLT3* inhibitors, such as midostaurin, quizartinib and gilteritinib [1, 2, 3].

A number of reviews report a prevalence of around 25% for *FLT3*‐ITD mutations in patients with AML and 5%–10% for *FLT3*‐TKD [1, 2, 3], but these do not reflect an objective summary assessment of the prevalence of *FLT3* mutations. A systematic literature review (SLR) of the prevalence of *FLT3* mutations in this disease has not been published. Thus, the purpose of the present study was to provide comprehensive information on this topic.

## 2 Materials and Methods

An SLR was carried out as per Preferred Reporting Items for Systematic Reviews and Meta‐Analyses guidelines [5] to identify literature reporting the prevalence of *FLT3* mutations in patients with AML. A study plan/protocol was developed; the study was not registered on a public register. Searches were conducted using Medline/PubMed and Embase literature databases through September 30, 2022; the earliest publication retrieved from our search was published in 1997. Based on these publications, data from the studies were generated between 1985 and 2021. Conference proceedings from the annual scientific meetings of the American Society of Hematology and European Haematology Association from 2012 to 2022 were also searched. Results were restricted to full‐text, English language articles only, but no limits were applied to geographical study location or publication year. Duplicate search results were excluded; this included duplicate publications, as well as multiple publications from the same dataset (in such instances, the publication with the largest dataset was included). Search terms used with these databases included medical subject headings, Embase terms, and free‐text words related to AML and *FLT3* mutations: ‘acute myeloid leukemia’, *‘AML’*, ‘*fms like tyrosine kinase 3*’, and *‘FLT3’* combined with *‘epidemiology’, ‘incidence’, ‘prevalence’, ‘frequency’, ‘presentation’*, and *‘occurrence’*. Medline/PubMed and Embase search strategies are summarised in Tables S1 and S2, respectively.

Inclusion and exclusion criteria (Table 1) were defined based on population, intervention, comparator, outcomes, and study design criteria [6] and applied to the titles and abstracts retrieved from the literature databases. Publications that passed abstract screening were retrieved in full‐text form and checked against the inclusion and exclusion criteria. Studies of < 200 patients were excluded due to lower estimate precision with smaller sample sizes and because an adequate number of larger samples were available.

**TABLE 1:** Inclusion and exclusion criteria.

PICOS criteria	Inclusion criteria	Exclusion criteria
Population	Paediatric and/or adult patients diagnosed with AML Sample size ≥ 200 patients Age range available Gender distribution available Geographical location available Source available (e.g., hospital) Dates of data collection available *FLT3* mutation test methodology provided	Patients diagnosed with acute promyelocytic leukaemia
Interventions	*Not relevant; no criteria assigned*	
Comparators	*Not relevant; no criteria assigned*	
Outcomes	*FLT3* mutation status (either *FLT3‐*ITD, *FLT3‐*TKD, or both)	Sample with only *FLT3* mutation positive or negative status (ITD or TKD not specified) No *FLT3* mutation status reported for the study population
Study design	Cross‐sectional studies Cohort studies Case–control studies Clinical trials	Review articles Interim analyses from clinical trials Animal studies In vitro studies In vivo studies Case reports Comments/commentaries News publications

Abbreviations: AML, acute myeloid leukaemia; *FLT3*, *fms‐like tyrosine kinase*; ITD, internal tandem duplication; PICOS, population, intervention, comparator, outcomes, and study design; SLR, systematic literature review; TKD, tyrosine kinase domain.

For each publication included in the SLR, relevant study and patient variables were extracted, recorded, and summarised with descriptive statistics (Table S3). Screening and data extraction were conducted by one investigator; a random sample of 25% of screened or extracted studies was evaluated for quality control by a second investigator knowledgeable of the protocol for the SLR. Extracted outcomes for the prevalence of the *FLT3* mutations were evaluated as a whole and by study type, geographic location of study, and patient age and gender.

To evaluate the heterogeneity of the included studies, an I^2^ index was calculated separately for those with populations drawn from interventional and non‐interventional studies [7, 8]. This study employed a descriptive approach to analyse the data and no formal hypothesis testing was conducted. The R (v4.1.3) package *meta* [9] was used to conduct meta‐analysis via random‐effects models, accounting for the heterogeneity of studies through a statistical parameter representing the inter‐study variation [10], to produce weighted summary estimates and confidence intervals (CIs) for the prevalence of each *FLT3* mutation from untransformed data.

## 3 Results

### 3.1 Screening Results

**FIGURE 1:**
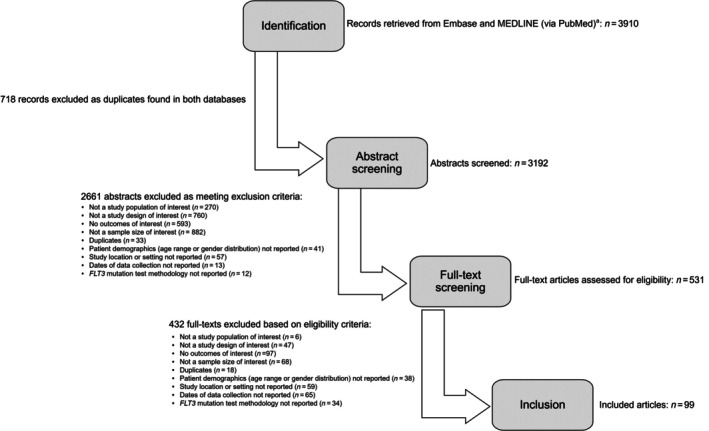
Literature identification and study selection process. ^a^Including conference abstracts. *FLT3*, *fms‐like tyrosine kinase 3*; SLR, systematic literature review.

In total, 3117 unique publications eligible for screening were obtained from the literature database searches (Figure 1); 2154 results were rejected during title and abstract screening and 841 during full‐text screening, and 99 publications were included in the SLR (Table S4). There was agreement between the first and second investigator across more than 95% of the studies in the random sample of screening and data extraction used for quality control.

### 3.2 Study Characteristics

For studies included in the SLR, dates of data collection ranged from 1985 to 2020 (Table 2). Sample sizes ranged from 201 to 3521 patients, with 54,135 patients included across all 99 studies. The majority of studies (77/99) used solely polymerase chain reaction (PCR) to identify *FLT3* mutations, with 22 mentions of alternative methods, namely gene sequencing and/or fluorescent fragment analysis.

**TABLE 2:** Characteristics of studies included in the SLR.

Characteristic	Studies including data on mutation prevalence	
	*FLT3*‐ITD (*n* = 99)	*FLT3*‐TKD (*n* = 42)^a^
Data collection period^b^, range	1985–2020	1986–2016
Sample size		
Range	201–3521	201–3521
Total	54,135	27,472
Method to detect *FLT3* mutation, *n*		
PCR	77	30
Gene sequencing	10	5
Fluorescent fragment analysis	2	1
PCR and fluorescent fragment analysis	3	2
Gene sequencing and fluorescent fragment analysis	1	0
PCR and gene sequencing	4	2
PCR, fluorescent fragment analysis, and gene sequencing	2	2
Study type, *n*		
Interventional	41	20
Non‐interventional	58	22
Geographical location, *n*		
Africa	2	0
Asia	33	14
Europe	45	22
Oceania	1	0
North America	10	3
South America	4	2
Multi‐continent	4	1
Age group of study population, *n*		
Paediatric (< 18 years) only	7	1
Younger adult (18–59 years) only	6	1
Older adult (≥ 60 years) only	6	2
Any adult (≥ 18 years)	33	13
Any age	47	25
Gender distribution of study population, % range		
Male	42.0–70.9	42.0–60.0
Female	29.1–58.0	40.0–58.0

Abbreviations: *FLT3*, *fms‐like tyrosine kinase 3*; ITD, internal tandem duplication; PCR, polymerase chain reaction; TKD, tyrosine kinase domain.

^a^ Included in the total number of studies reporting *FLT3*‐ITD data.

^b^ Data collection period refers to the data included in the original studies.

### 3.3 Prevalence of *FLT3* Mutations

**FIGURE 2:**
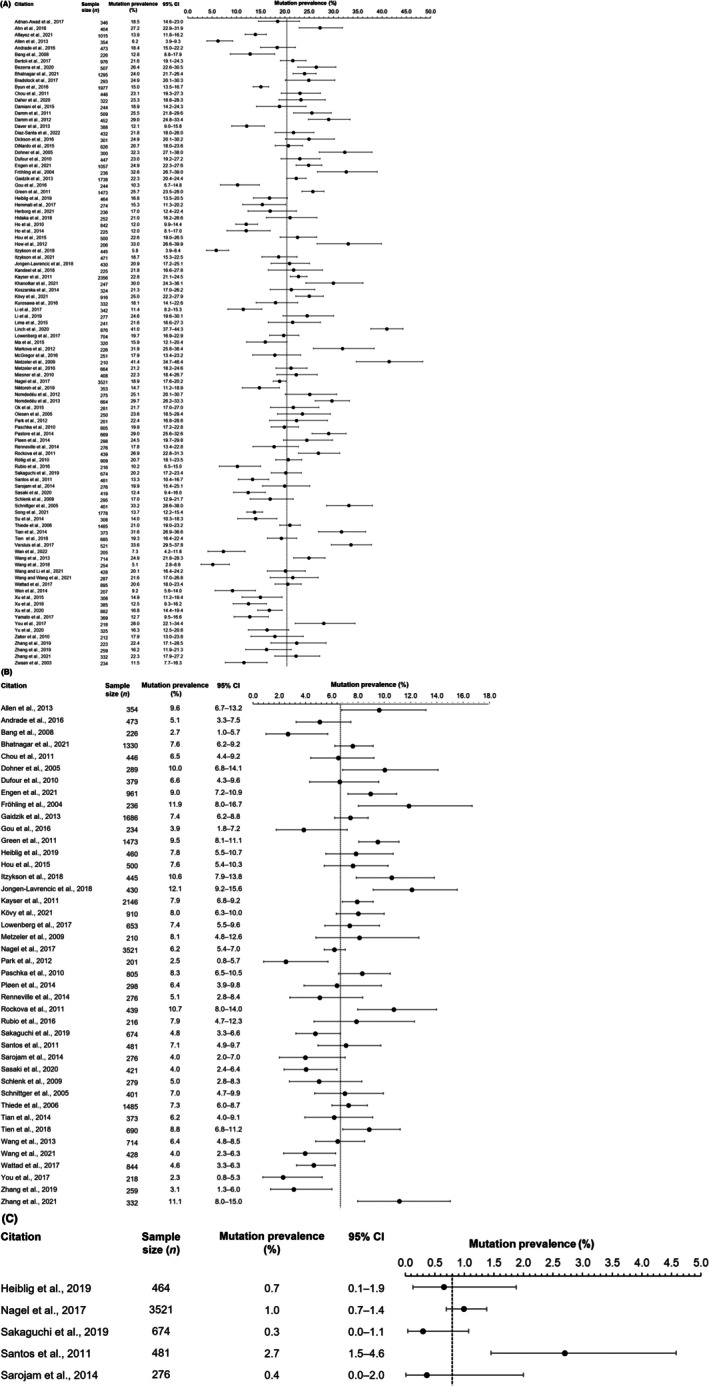
Distribution of *FLT3*‐ITD mutation (A), *FLT3*‐TKD mutation (B), and *FLT3*‐ITD/*FLT3*‐TKD co‐mutation (C) prevalence estimates reported in studies included in the SLR. Error bars denote 95% CIs. Dashed vertical line denotes weighted mean prevalence of mutation calculated in the present study. Full citations can be found in Supplementary References. CI, confidence interval; *FLT3*, *fms‐like tyrosine kinase 3*; ITD, internal tandem duplication; SLR, systematic literature review; TKD, tyrosine kinase domain.

The weighted mean prevalence of *FLT3*‐ITD mutations (20.4%; 95% CI: 19.1%–21.8%) was higher than that of *FLT3*‐TKD mutations (6.8%; 95% CI: 6.1%–7.5%) or *FLT3*‐ITD/*FLT3*‐TKD co‐mutations (0.8%; 95% CI: 0.2%–1.4%) (Table 3; Figure 2A–C). The range of prevalence estimates across individual studies was 5.1%–41.4% for *FLT3*‐ITD and 2.3%–12.1% for *FLT3*‐TKD mutations (Figure 2A,B).

**TABLE 3:** Prevalence of *FLT3*‐ITD and *FLT3*‐TKD mutations and co‐mutations reported in studies included in the SLR.

Group	Mutation								
	*FLT3*‐ITD			*FLT3*‐TKD^a^			Co‐mutation^b^		
	*n*	Range of prevalence	Weighted mean prevalence (95% CI)	*n*	Range of prevalence	Weighted mean prevalence (95% CI)	*n*	Range of prevalence	Weighted mean prevalence (95% CI)
All studies	99	5.12–41.43	20.43 (19.05–21.81)	42	2.29–12.09	6.79 (6.06–7.53)	5	0.30–2.70	0.80 (0.22–1.38)
Study design									
Non‐interventional	58	5.12–33.01	19.22 (17.69–20.75)	22	2.29–11.14	5.62 (4.17–6.54)	5	0.30–2.70	0.80 (0.22–1.38)
Interventional	41	5.84–41.43	22.06 (19.58–24.53)	20	4.62–12.09	8.03 (7.18–8.88)	0	—	—
Geographical location									
Asia	33	5.12–31.64	17.85 (15.75–19.95)	14	2.29–11.14	5.16 (3.87–6.44)	2	0.30–0.36	0.31 (0.00–0.67)
Europe	45	5.84–41.43	23.18 (21.19–25.16)	22	4.62–12.09	7.80 (7.02–8.57)	2	0.65–0.99	0.94 (0.64–1.23)
North America	10	12.11–33.01	20.12 (15.66–24.59)	3	4.04–7.59	6.26 (4.04–8.47)	1	—	—
South America	4	10.19–26.43	19.12 (12.38–25.86)	2	5.07–7.87	6.05 (3.44–8.67)	0	—	—
Age range^c^									
Paediatric (< 18 years) only	7	5.12–16.78	11.79 (8.87–14.71)	1	—	—	0	—	—
Younger adult (18–59 years) only	6	15.33–28.98	22.77 (19.13–26.41)	1	—	—	0	—	—
Older adult (≥ 60 years) only	6	12.11–24.92	18.21 (14.90–21.53)	2	5.02–7.83	6.45 (3.70–9.20)	1	—	—
Any adult (≥ 18 years)	33	12.41–41.43	21.75 (19.67–23.83)	13	3.99–12.09	6.40 (5.39–7.41)	3	0.36–2.70	1.20 (0.01–2.40)
Any age	47	5.84–40.98	20.78 (18.60–22.96)	25	2.29–11.86	6.97 (5.87–8.08)	1	—	—
Gender									
Male	14	5.84–33.04	18.19 (14.69–21.68)	5	4.38–6.03	5.39 (4.54–6.24)	—	—	—
Female	14	8.00–36.09	21.51 (17.10–25.92)	5	2.92–10.34	5.35 (3.36–7.35)	—	—	—

Abbreviations: –, no data available; CI, confidence interval; *FLT3, fms‐like tyrosine kinase 3*; SLR, systematic literature review.

^a^ Included in the total number of studies reporting *FLT3*‐ITD data.

^b^ *FLT3*‐ITD and *FLT3*‐TKD.

^c^ Age ranges in the studies fell largely into these categories.

*FLT3* gene mutations have been reported from a spectrum of cytogenetic groups. Fourteen studies included only patients diagnosed with cytogenetically normal AML (CN‐AML), with the range in prevalence of *FLT3*‐ITD mutations in these patients being 7.3%–41.4% [11, 12, 13, 14, 15, 16, 17, 18, 19, 20, 21, 22, 23, 24]. Across 18 studies, the prevalence of *FLT3*‐ITD and nucleophosmin (*NPM1*) co‐mutations was 0.9%–28.0% among AML patients, with the prevalence of *NPM1* mutations ranging from 9.0%–60.0% among patients with *FLT3*‐ITD mutations [11, 13, 14, 17, 25, 26, 27, 28, 29, 30, 31, 32, 33, 34, 35, 36, 37, 38].

A total of 41 studies were interventional versus 58 non‐interventional (Tables 2 and 3). The weighted mean prevalence estimates for *FLT3‐*ITD and *FLT3‐*TKD mutations were slightly higher in patient populations from interventional studies compared with those in patient populations from non‐interventional studies (Table 3). For interventional studies, the I^2^ indices were 96% and 68% among studies reporting *FLT3*‐ITD and *FLT3*‐TKD mutations, respectively. For non‐interventional studies, the I^2^ indices were 89% and 74% among studies reporting *FLT3*‐ITD and *FLT3*‐TKD mutations, respectively.

The majority of studies were conducted in Asia and Europe (Tables 2 and 3). No studies specifically analysed the prevalence of *FLT3* mutations by geographic location. The weighted means for the prevalence of the *FLT3*‐ITD mutations among AML patients tended to be higher for study populations from Europe, followed by North America, South America, and Asia (Table 3). Similarly, the weighted means for the prevalence of the *FLT3*‐TKD mutations among AML patients tended to be higher for study populations from Europe than those from North America or Asia, but there was no available prevalence of this mutation in study populations from South America.

Seven studies included paediatric patients (aged < 18 years) only; 6 were restricted to younger adults (aged 18–59 years) and 6 to older adults (aged ≥ 60 years) only; 33 included adult patients of any age; and 47 included patients of any age (Tables 2 and 3). Prevalence of *FLT3* mutations was stratified by age in eight studies. Prevalence of *FLT3*‐ITD mutations tended to be higher in younger adults and lower in paediatric and older populations (Table 3).

Across studies, 42.0%–70.9% of participants were male (Table 2), with 14 studies stratifying the prevalence of *FLT3* mutations by gender. The range of prevalence estimates among both males and females was wide and, in the majority of studies, the prevalence of *FLT3*‐ITD mutations was higher in females than in males but was similar for *FLT3*‐TKD mutations (Table 3).

## 4 Discussion

In the present study, the global prevalence of *FLT3* mutations among patients with AML was evaluated in the published literature via SLR and meta‐analyses. The weighted mean *FLT3*‐ITD mutation prevalence estimated for this patient population was 20.4% (95% CI: 19.1%–21.8%), lower than the estimate referenced in several recent reviews of AML treatment (approximately 25.0%) [1, 2, 3]. In contrast, the weighted mean prevalence for the *FLT3*‐TKD mutation (6.8%; 95% CI: 6.1%–7.5%) fell within the range included in these previous reviews (5.0%–10.0%) [1, 2, 3]. Of note, there was wide variability in mutation prevalence estimates between the studies (*FLT3*‐ITD: 5.1%–41.4%; *FLT3*‐TKD: 2.3%–12.0%), the reason for which is poorly understood and cannot be explained by the population‐based factors described. Moreover, for four of the six primary publications referenced in the above reviews [39, 40, 41, 42], their *FLT3* mutation prevalence estimates were not included in our SLR due to our sample size requirements (≥ 200 patients) or because their title and/or abstract lacked our predefined search terms, highlighting a potential limitation of our methodology.

The prevalence of the *FLT3*‐ITD mutation among patients diagnosed with CN‐AML ranged from 14.9%–41.4%, with previous reports ranging from 33.0%–37.0% [43, 44]. One reason for this difference may be that we assessed *FLT3*‐ITD mutation prevalence globally and across all ages, whereas some of the previous studies that reported higher *FLT3*‐ITD mutation prevalence focused on European populations or on patients under 60 years old [43, 44]. Moreover, there was a wide range of prevalence estimates for *FLT3*‐ITD and *NPM1* co‐mutations in AML, from 0.9%–28.0%, with the prevalence of the *NPM1* mutation among patients with known *FLT3*‐ITD mutations ranging from 9.0%–60.0%. Importantly, the European LeukemiaNet 2022 prognostic classification considers *FLT3*‐ITD mutations to supersede *NPM1* mutations, such that patients with *FLT3*‐ITD are classified as intermediate risk regardless of FLT3‐*ITD* allelic ratio or *NPM1* co‐mutation [45]. The weighted mean prevalence estimates for the *FLT3‐*ITD and *FLT3‐*TKD mutations were slightly elevated in patient populations from interventional studies (*FLT3‐*ITD: 22%; *FLT3‐*TKD: 8%) compared with those from non‐interventional studies (*FLT3‐*ITD: 19%; *FLT3‐*TKD: 6%). This previously unreported finding is likely to be explained by differences in patient selection: non‐interventional studies tend to reflect a broader patient population with less restricted inclusion and exclusion criteria [46]. The results of the heterogeneity tests for the interventional and non‐interventional studies were > 50%, suggesting that differences in results were due to a high level of variability between studies, rather than sampling error.

When grouped by geographic location, weighted mean *FLT3* mutation prevalence estimates tended to be higher for Europe (*FLT3‐*ITD: 23%; *FLT3‐*TKD: 8%) and lower for Asia (*FLT3‐*ITD: 18%; *FLT3‐*TKD: 5%). As previously mentioned, our SLR inclusion criteria required study populations ≥ 200 patients, and this resulted in a disproportionate exclusion of studies from regions other than Europe and Asia. Moreover, to date no studies have specifically analysed the prevalence of *FLT3* mutations by geographic location, highlighting a gap in the literature.

Younger adults (aged 18–59 years) tended to have higher weighted mean prevalence of *FLT3‐*ITD mutations (23%) of all age groups evaluated, with paediatric (aged < 18 years) and older (aged ≥ 60 years) populations having lower mean prevalence (12% and 18%, respectively). This is consistent with a study included in the SLR, which determined that paediatric patients had a significantly lower prevalence of *FLT3*‐ITD mutations than adults [47]. It is important to note that there were considerably fewer studies evaluating *FLT3* mutation prevalence in patients under 60 years, which could be attributable to the fact that AML predominantly affects the elderly [48].

The weighted mean prevalence of *FLT3*‐ITD mutations was higher in females (22%) than in males (18%). This was consistent with two of the studies in the SLR, in which the proportions of *FLT3* mutations were significantly higher in females than in males [31, 49]. This also aligns with two phase 3 clinical trials evaluating treatments for patients with newly diagnosed or relapsed/refractory *FLT3*‐mutated AML, both of which enrolled more female than male patients [50, 51].

The present study is the first comprehensive SLR focused on the global reported prevalence of *FLT3* mutations among AML patients and features a robust compilation of over 50,000 patients with *FLT3*‐mutated AML, considerably larger than the largest previous study, which was a registry study of approximately 3500 patients (and has been included in this SLR) [49].

With regard to limitations, we did not undertake a pre‐planned analysis to evaluate differences in *FLT3* mutation prevalence by mutational testing method, and differences in accuracy between these methods may impact mutation prevalence estimates [52, 53]. However, from a post hoc analysis (Table S5), there was no suggestion that testing methodologies (PCR vs. non‐PCR) were associated with systematic differences in *FLT3* mutation prevalence for study type or geographic region. Additionally, few studies included in the SLR reported the prevalence of the *FLT3* mutations at different stages of disease progression, which is important to understand considering that *FLT3* mutations can dynamically either be lost or arise at different stages of AML treatment and progression [3]. Lastly, as previously mentioned, heterogeneity among both interventional and non‐interventional studies was high, and prevalence estimates of individual studies varied widely.

## 5 Conclusion

In conclusion, the results of this SLR and meta‐analysis of global *FLT3* mutation frequency in patients with AML indicate a global population prevalence of 20% for *FLT3*‐ITD mutations and 7% for *FLT3*‐TKD. Although no formal hypothesis testing was conducted, our findings provide valuable insights into the distribution of *FLT3* mutations that could inform future research directions and clinical trials. However, further work is needed to understand the considerable heterogeneity in reported study‐level prevalence estimates to more accurately identify patients harbouring *FLT3* mutations, and to determine how *FLT3* mutations differ across age, sex, geography and stage of disease progression.

## Author Contributions

**Juliana F. M. Lewis:** conceptualization, methodology, investigation, data curation, validation, formal analysis, writing – review and editing. **Naval G. Daver:** conceptualization, methodology, formal analysis, writing – review and editing. **Noah Jamie Robinson:** conceptualization, methodology, formal analysis, writing – review and editing. **Bhavik J. Pandya:** conceptualization, methodology, formal analysis, writing – review and editing. **Bosny Pierre‐Louis:** formal analysis, writing – review and editing. **Sayma Monir:** investigation, data curation, validation, writing – review and editing. **Jorge Sierra:** conceptualization, methodology, formal analysis, writing – review and editing.

## Conflicts of Interest

J.F.M.L., N.J.R., B.J.P., B.P.‐L. and S.M. are employees of Astellas. N.G.D. and J.S. have nothing relevant to disclose.

## Supporting information


**Table S1:** Medline/PubMed search algorithm.
**Table S2:** Embase search algorithm.
**Table S3:** Patient and study variables extracted from studies included in the SLR.
**Table S4:** Characteristics of studies included in the SLR.
**Table S5:** Prevalence of *FLT3*‐ITD and *FLT3*‐TKD mutations by testing method, across study design and geographic location.

## Data Availability

The data used in this systematic literature review were extracted from the existing studies cited in the manuscript, some of which are available in the public domain with others requiring a fee for access. The data extracted from each study are described in Table S4.
