# Supplementary material for: Prevalence of Fms‐Like Tyrosine Kinase 3 (FLT3) Mutations in Patients With Acute Myeloid Leukaemia: A Systematic Literature Review and Meta‐Analysis
Source: Cancer Med. 2025 Sep 22;14(18):e71205. doi: 10.1002/cam4.71205 (PMC12451824; doi:10.1002/cam4.71205)
Supplement: Supplementary file 1 — Table S1: Medline/PubMed search algorithm. Table S2: Embase search algorithm. Table S3: Patient and study variables extracted from studies included in the SLR. Table S4: Characteristics of studies included in the SLR. Table S5: Prevalence of FLT3‐ITD and FLT3‐TKD mutations by testing method, across study design and geographic location. [file CAM4-14-e71205-s001.docx]

**Supplementary Tables**

**Supplementary Table 1. Medline/PubMed search algorithm**

| **Search string number** | **Search algorithm** |
| --- | --- |
| #1 | MeSH ((acute myeloid leukemia) OR “acute myeloid leukemia” OR AML) |
| #2 | MeSH ((fms like tyrosine kinase 3) OR (fms like tyrosine kinase 3 mutat*) OR (FLT3 mutat*) OR (fms like tyrosine kinase 3) OR FLT3 OR (fms like tyrosine kinase 3)) |
| #3 | MeSH (epidemiology OR incidence OR prevalence OR frequency OR presentation OR occurrence) |
| #4 | #1 AND #2 AND #3 AND [English]/lim |
| #5 | #4 NOT (animals OR editorial[Publication type] OR news[Publication type] OR case reports[Publication type]) |

*Searches all terms that have the root. AML, acute myeloid leukemia; *FLT3, fms-like tyrosine kinase 3*; MeSH, Medical Subject Headings.

**Supplementary Table 2. Embase search algorithm**

| **Search string** | **Search algorithm** |
| --- | --- |
| #1 | (EMB (acute myeloid leukemia) OR “acute myeloid leukemia” OR AML) |
| #2 | (EMB (fms like tyrosine kinase 3) OR (fms like tyrosine kinase 3 mutat*) OR (FLT3 mutat*) OR (fms like tyrosine kinase 3) OR FLT3 OR (fms like tyrosine kinase 3)) |
| #3 | (EMB (epidemiology OR incidence OR prevalence OR frequency OR presentation OR occurrence) |
| #4 | #1 AND #2 AND #3 AND [English]/lim |
| #5 | #4 NOT (“animal”/exp OR “animal model”/de OR “case report”/de OR “editorial”/de OR “news”/de) |

*Searches all terms that have the root. AML, acute myeloid leukemia; EMB, Embase; *FLT3, fms-like tyrosine kinase 3*.

**Supplementary Table 3. Patient and study variables extracted from studies included in the SLR**

| **Patient variable** | **Study variable** |
| --- | --- |
| - Type of AML - Severity of AML (i.e., relapsed/refractory) - Type of karyotype - Age range of study population - Gender distribution of study population - Type of co-mutations (non-*FLT3)* | - Publication type (abstract; full text) - First author; publication year - Study name (if applicable) - Study design - Geographical location (continent; country) - Name and number of study centers - Years of data collection - Sample size at baseline - Source of study sample - Description of *FLT3* mutation test methodology |

AML, acute myeloid leukemia; *FLT3, fms-like tyrosine kinase 3*; SLR, systematic literature review.

**Supplementary Table 4. Characteristics of studies included in the SLR**

| **Number** | **First author surname** | **Publication year** | **Data collection period** | **Sample size** | ***FLT3* mutation detection method** | ***FLT3* mutation type*** | | **Study type** | **Geographical location** | **Patient age range, years** | **Percentage of males** |
| --- | --- | --- | --- | --- | --- | --- | --- | --- | --- | --- | --- |
|  |  |  |  |  |  | **ITD** | **TKD** |  |  |  |  |
| 1 | Adnan-Awad | 2017 | 2011–2015 | 346 | RT-PCR | X |  | Non-interventional | Africa | 14–83 | 47.1 |
| 2 | Ahn | 2016 | 1998–2012 | 404 | PCR, Sanger sequencing | X |  | Non-interventional | Asia | 15–84 | 49.3 |
| 3 | Alfayez | 2021 | 2013–2017 | 1406 | NGS | X |  | Non-interventional | North America | 17–93 | 57.5 |
| 4 | Allen | 2013 | 1988–2009 | 354 | PCR | X | X | Interventional | Europe; Oceania | 15–59 | 56.0 |
| 5 | Andrade | 2016 | 2000–2015 | 703 | PCR | X | X | Non-interventional | South America | 0–21 | 54.9 |
| 6 | Bang | 2008 | 1996–2005 | 226 | PCR | X | X | Non-interventional | Asia | 0–86 | 52.2 |
| 7 | Bertoli | 2017 | 2000–2014 | 976 | PCR | X |  | Non-interventional | Europe | 36.6–72 | 51.9–60.5 |
| 8 | Bezerra | 2020 | 2003–2019 | 507 | PCR | X |  | Non-interventional | South America | 18–94 | 47.0 |
| 9 | Bhatnagar | 2021 | 1986–2015 | 1339 | Amplicon sequencing; gene sequencing | X | X | Interventional | North America | 17–92 | 55.1 |
| 10 | Bradstock | 2017 | 2003–2010 | 293 | PCR | X |  | Interventional | Oceania | 16–60 | 56.0 |
| 11 | Byun | 2016 | 2007–2011 | 1977 | PCR | X |  | Non-interventional | Asia | 15–88 | 54.5 |
| 12 | Chou | 2011 | 1995–2007 | 446 | PCR | X | X | Non-interventional | Asia | 18–90 | 56.3 |
| 13 | Daher | 2020 | 2015–2018 | 435 | PCR | X |  | Non-interventional | North America | 18–82 | 53.3 |
| 14 | Damiani | 2015 | 2008–2014 | 244 | RT-PCR | X |  | Non-interventional | Europe | 18–84 | 70.9 |
| 15 | Damm | 2011 | 1995–2004 | 509 | RT-PCR | X |  | Interventional | Europe | 17–60 | 52.0 |
| 16 | Damm | 2012 | 1999–2004 | 452 | RT-PCR | X |  | Interventional | Europe | 17–60 | 45.0–53.0 |
| 17 | Daver | 2013 | 2000–2010 | 557 | PCR | X |  | Non-interventional | North America | 65–89 | 64.0 |
| 18 | Diaz-Santa | 2022 | 2004–2017 | 477 | PCR | X |  | Interventional | Europe | 15–71 | 52.6 |
| 19 | Dickson | 2016 | 1990–1998 | 301 | PCR | X |  | Interventional | Europe | 60–85 | 56.5 |
| 20 | DiNardo | 2015 | 2010–2014 | 826 | PCR | X |  | Non-interventional | North America | 18–92 | 56.9 |
| 21 | Dohner | 2005 | 1993–2004 | 300 | PCR | X | X | Interventional | Europe | 16–60 | 43.7 |
| 22 | Dufour | 2010 | 1999–2007 | 467 | PCR;  RT-PCR | X | X | Interventional | Europe | 17–85 | 53.7 |
| 23 | Engen | 2021 | 1987–2013 | 1057 | Fragment analysis; PCR; Sanger sequencing | X | X | Interventional | Europe | 15–80 | 53.8 |
| 24 | Fröhling | 2004 | 1993–2002 | 236 | PCR | X | X | Interventional | Europe | 16–60 | 45.8 |
| 25 | Gaidzik | 2013 | 1998–2013 | 1770 | PCR | X | X | Interventional | Europe | 18–60 | 51.2 |
| 26 | Gou | 2016 | 2012–2014 | 255 | PCR | X | X | Non-interventional | Asia | 15–80 | 54.0 |
| 27 | Green | 2011 | 1988–2002 | 1473 | PCR | X | X | Interventional | Europe | 15–68 | 49.0 |
| 28 | Heiblig | 2019 | 2000–2016 | 495 | PCR | X | X | Non-interventional | Europe | 64–93 | 60.0 |
| 29 | Hemmati | 2017 | 2002–2008 | 274 | PCR | X |  | Interventional | Europe | 18 –60 | 52.2 |
| 30 | Herborg | 2021 | 2009–2018 | 268 | NGS | X |  | Non-interventional | Europe | 19–94 | 53.4 |
| 31 | Hidaka | 2018 | 2007–2017 | 252 | PCR; electrophoresis | X |  | Non-interventional | Asia | 17–89 | 56.0 |
| 32 | Ho | 2010 | 1995–2005 | 842 | PCR | X |  | Interventional | Europe North America Oceania | 0.85–21.63 | 53.2 |
| 33 | Ho | 2014 | 2003–2005 | 225 | PCR | X |  | Interventional | Europe North America Oceania | 0.1–20.8 | 56.0 |
| 34 | Hou | 2015 | 1995–2008 | 500 | PCR | X | X | Non-interventional | Asia | 15–90 | 57.0 |
| 35 | How | 2012 | 2002–2010 | 206 | PCR | X |  | Non-interventional | North America | 21–77 | 54.4 |
| 36 | Itzykson | 2018 | 2005–2015 | 445 | PCR | X | X | Interventional | Europe | 1–81 | 56.4 |
| 37 | Itzykson | 2021 | 2012–2016 | 471 | Targeted sequencing; fragment analysis | X |  | Interventional | Europe | 60–85 | 56.7 |
| 38 | Jongen-Lavrencic | 2018 | 2001–2013 | 430 | NGS | X | X | Interventional | Europe | 18–66 | 50.0 |
| 39 | Kandeel | 2018 | 2011–2014 | 257 | PCR; electrophoresis | X |  | Non-interventional | Africa | 18–60 | 53.3 |
| 40 | Kayser | 2011 | 1993–2008 | 2853 | PCR | X | X | Interventional | Europe | 16.2–85.0 | 51.6 |
| 41 | Khanolkar | 2021 | 2008–2020 | 247 | PCR | X |  | Non-interventional | North America | 18–72 | 47.0 |
| 42 | Koszarska | 2014 | 2001–2009 | 324 | PCR | X |  | Non-interventional | Europe | 16–60 | 46.3 |
| 43 | Kövy | 2021 | 2001–2020 | 916 | As per ELN guidelines^†^ | X | X | Non-interventional | Europe | 16–94 | 49.0 |
| 44 | Kurosawa | 2016 | 1999–2010 | 332 | PCR | X |  | Non-interventional | Asia | 16–70 | 59.0 |
| 45 | Li | 2017 | 2005–2013 | 342 | PCR | X |  | Interventional | Asia | 1–16 | 60.8 |
| 46 | Li | 2019 | 2013–2016 | 277 | Sanger sequencing | X |  | Non-interventional | Asia | 8–73 | 57.4 |
| 47 | Lima | 2015 | 2005–2015 | 241 | PCR | X |  | Non-interventional | South America | 18–97 | 47.0 |
| 48 | Linch | 2020 | 1988–2014 | 876 | PCR | X |  | Interventional | Europe | 16–59 | 42.0 |
| 49 | Lowenberg | 2017 | 2010–2013 | 795 | Targeted sequencing; electrophoresis | X | X | Interventional | Europe | 18–65 | 55.8 |
| 50 | Ma | 2015 | 2007–2013 | 320 | WGS; RT-PCR | X |  | Non-interventional | Asia | 16–85 | 58.4 |
| 51 | Markova | 2012 | 1994–2010 | 226 | RT-PCR | X |  | Non-interventional | Europe | 18.2–81.7 | 47.3 |
| 52 | McGregor | 2016 | 2007–2011 | 363 | PCR; Fragment analysis | X |  | Non-interventional | Europe | 19–93 | 52.0 |
| 53 | Metzeler | 2009 | 1999–2004 | 210 | PCR | X | X | Interventional | Europe | 17–83 | 42.0 |
| 54 | Metzeler | 2016 | 1999–2012 | 664 | PCR | X |  | Interventional | Europe | 18–86 | 50.3 |
| 55 | Miesner | 2010 | 2005–2009 | 408 | PCR | X |  | Non-interventional | Europe | 18.3–88.1 | 52.0 |
| 56 | Nagel | 2017 | 2012–2014 | 3521 | PCR; direct sequencing; fragment analysis | X | X | Non-interventional | Europe | 18–94 | 55.1 |
| 57 | Niktoreh | 2019 | 2004–2017 | 353 | RT-PCR | X |  | Interventional | Europe | 0–18 | 51.8 |
| 58 | Nomdedéu | 2012 | 2004–2010 | 275 | PCR | X |  | Interventional | Europe | 17–73 | 58.5 |
| 59 | Nomdedéu | 2013 | 2004–2011 | 664 | PCR | X |  | Interventional | Europe | 16–70 | 55.1 |
| 60 | Ok | 2015 | 2012–2013 | 281 | NGS | X |  | Non-interventional | North America | 1–92 | 58.7 |
| 61 | Olesen | 2005 | 1985–2002 | 250 | RT-PCR | X |  | Non-interventional | Europe | 16–91 | 42.4 |
| 62 | Park | 2012 | 2002–2010 | 201 | PCR | X | X | Non-interventional | Asia | 15–81 | 50.7 |
| 63 | Paschka | 2010 | 1998–2004 | 805 | PCR | X | X | Interventional | Europe | 16–60 | 50.9 |
| 64 | Pastore | 2014 | 1999–2005 | 669 | PCR | X |  | Interventional | Europe | 17–85 | 49.8 |
| 65 | Pløen | 2014 | 2005–2013 | 298 | Pyrosequencing; fragment analysis | X | X | Non-interventional | Europe | 17–98 | 51.7 |
| 66 | Renneville | 2014 | 2008–2010 | 278 | PCR | X | X | Interventional | Europe | 50–70 | 50.0 |
| 67 | Rockova | 2011 | 1987–2006 | 439 | PCR | X | X | Interventional | Europe | 15–60 | 50.1 |
| 68 | Röllig | 2010 | 1996–2004 | 909 | PCR | X |  | Interventional | Europe | 61–87 | 51.6 |
| 69 | Rubio | 2016 | 2000–2014 | 216 | PCR | X | X | Non-interventional | South America | 0–17.9 | 53.7 |
| 70 | Sakaguchi | 2019 | 2000–2018 | 674 | PCR-RFLP | X | X | Non-interventional | Asia | 15–94 | 58.6 |
| 71 | Santos | 2011 | 2003–2007 | 481 | PCR | X | X | Non-interventional | North America | 17–85 | 51.6 |
| 72 | Sarojam | 2014 | 2009–2011 | 276 | PCR; PCR-RFLP | X | X | Non-interventional | Asia | 18–74 | 51.4 |
| 73 | Sasaki | 2020 | 2013–2016 | 421 | NGS; PCR | X | X | Non-interventional | North America | 17–92 | 58.0 |
| 74 | Schlenk | 2009 | 1998–2004 | 377 | PCR | X | X | Interventional | Europe | 61–84 | 54.6 |
| 75 | Schnittger | 2005 | 1999–2004 | 401 | PCR; RT-PCR; gene sequencing | X | X | Interventional | Europe | 16.8–81.9 | 50.4 |
| 76 | Song | 2021 | 2006–2012 | 2110 | PCR | X |  | Non-interventional | Asia | 18–95 | 54.3 |
| 77 | Su | 2014 | 2009–2013 | 312 | PCR | X |  | Non-interventional | Asia | 7–82 | 52.2 |
| 78 | Thiede | 2006 | 1996–2003 | 1485 | PCR | X | X | Interventional | Europe | 15–87 | 52.4 |
| 79 | Tian | 2014 | 2005–2010 | 373 | PCR | X | X | Non-interventional | Asia | 15–83 | 52.8 |
| 80 | Tien | 2018 | 1994–2011 | 693 | PCR | X | X | Non-interventional | Asia | 15–94 | 57.0 |
| 81 | Versluis | 2017 | 1995–2010 | 521 | RT-PCR | X |  | Interventional | Europe | 16–60 | 50.3 |
| 82 | Wan | 2022 | 2013–2017 | 205 | PCR | X |  | Non-interventional | Asia | 11–65 | 57.6 |
| 83 | Wang | 2013 | 2005–2010 | 714 | PCR | X | X | Non-interventional | Asia | 8–83 | 55.5 |
| 84 | Wang | 2018 | 2007–2017 | 254 | PCR | X |  | Non-interventional | Asia | 0.2–16 | 56.3 |
| 85 | Wang | 2021 | 2006–2016 | 428 | PCR | X | X | Non-interventional | Asia | 16–73 | 51.6 |
| 86 | Wang | 2021 | 2015–2017 | 287 | NGS | X |  | Non-interventional | Asia | 11–79 | 58.5 |
| 87 | Wattad | 2017 | 1993–2009 | 1025 | PCR | X | X | Interventional | Europe | 18.0–84.5 | 57.6 |
| 88 | Wen | 2014 | 2008–2014 | 233 | PCR | X |  | Non-interventional | Asia | 15–93 | 57.9 |
| 89 | Xu | 2015 | 2005–2014 | 308 | PCR | X |  | Non-interventional | Asia | 16–68 | 46.8 |
| 90 | Xu | 2018 | 2004–2016 | 460 | PCR, Sanger sequencing | X |  | Non-interventional | Asia | 18–86 | 56.5 |
| 91 | Xu | 2020 | 1996–2010 | 885 | PCR | X |  | Interventional | Europe North America Oceania | <3 – <18 | 52.2 |
| 92 | Yamato | 2017 | 2006–2010 | 369 | PCR | X |  | Interventional | Asia | 0 to <18 | 48.1 |
| 93 | You | 2017 | 2008–2015 | 219 | PCR | X | X | Non-interventional | Asia | 1–84 | 55.7 |
| 94 | Yu | 2020 | 2016–2019 | 325 | PCR | X |  | Non-interventional | Asia | 16–87 | 53.8 |
| 95 | Zaker | 2010 | 2006–2007 | 212 | PCR | X |  | Non-interventional | Asia | 18–75 | 59.4 |
| 96 | Zhang | 2019 | 2010–2017 | 223 | PCR | X |  | Non-interventional | Asia | 17–60 | 47.1 |
| 97 | Zhang | 2019 | 2014–2016 | 259 | Sanger sequencing | X | X | Non-interventional | Asia | 2–68 | 59.1 |
| 98 | Zhang | 2021 | 2010–2018 | 332 | Targeted-capture sequencing | X | X | Non-interventional | Asia | 12–60 | 60.2 |
| 99 | Zwaan | 2003 | 1990–2001 | 234 | PCR, RT-PCR | X |  | Interventional | Europe | 0–18 | 53.8 |

*X denotes mutation was detected in patient sample. ^†^Döhner H, et al. Diagnosis and management of AML in adults: 2017 ELN recommendations from an international expert panel. *Blood*. 2017;129(4):424-447.

DNA, deoxyribonucleic acid; ELN, European LeukemiaNet; *FLT3, fms-like tyrosine kinase 3*; ITD, internal tandem deletion; NGS, next-generation sequencing; PCR, polymerase chain reaction; RFLP, restriction fragment length polymorphism; RT, reverse transcription; SLR, systematic literature review; TKD, tyrosine kinase domain; WGS, whole genome sequencing.

**Supplementary Table 5. Prevalence of *FLT3*-ITD and *FLT3*-TKD mutations by testing method, across study design and geographic location**

|  | ***FLT3-ITD*** | | | | ***FLT3-TKD*** | | | | |
| --- | --- | --- | --- | --- | --- | --- | --- | --- | --- |
|  | **PCR Test** | | **Non-PCR Test** | | **PCR Test** | | **Non-PCR Test** | | |
|  | **n** | **Weighted mean prevalence**  **(95% CI)** | **n** | **Weighted mean prevalence**  **(95% CI)** | **n** | **Weighted mean prevalence**  **(95% CI)** | **n** | **Weighted mean prevalence**  **(95% CI)** |  |
| **Study design** | | | | | | | | |  |
| Non-interventional | 42 | 19.32 (17.36 - 21.28) | 16 | 19.09 (17.01 – 21.18) | 15 | 5.72 (4.58 – 6.86) | 7 | 5.43 (3.78 – 7.07) |  |
| Interventional | 35 | 21.83 (19.02 – 24.65) | 6 | 23.44 (19.48 – 27.40) | 15 | 7.92 (6.87 – 8.96) | 5 | 8.34 (6.94 – 9.73) |  |
| **Geographical location** | | | | | | | | |  |
| Asia | 24 | 17.08 (14.48 – 19.68) | 9 | 19.90 (16.85 -22.96) | 10 | 5.06 (3.61 – 6.50) | 4 | 5.54 (2.23 – 8.85) |  |
| Europe | 36 | 23.56 (21.20 – 25.92) | 9 | 21.66 (18.49 – 24.83) | 16 | 7.82 (6.89 – 8.75) | 6 | 7.78 (6.22 – 9.35) |  |
| Americas* | 10 | 20.62 (16.00 – 25.24) | 4 | 18.52 (13.28 – 23.75) | 3 | 6.33 (4.64 – 8.02) | 2 | 5.87 (2.39 – 9.35) |  |

^*^Americas = North America and South America.

CI, confidence interval; FLT3, fms-like tyrosine kinase 3; ITD, internal tandem duplication; PCR, polymerase chain reaction; TKD, tyrosine kinase domain.

**Supplementary References**

List of all papers included in the SLR:

1. Adnan-Awad S, Gaber O, Eltokhy SA, et al. FLT3-ITD mutations in Egyptian patients of acute myeloid leukemia: correlation with cytogenetic, FAB subgroups and prognosis. *Clin Lab*. 2017;63(5):1027-1034.
2. Ahn JS, Kim JY, Kim HJ, et al. Normal karyotype acute myeloid leukemia patients with CEBPA double mutation have a favorable prognosis but no survival benefit from allogeneic stem cell transplant. *Ann Hematol*. 2016;95(2):301-310.
3. Alfayez M, Issa GC, Patel KP, et al. The clinical impact of PTPN11 mutations in adults with acute myeloid leukemia. *Leukemia*. 2021;35(3):691-700.
4. Allen C, Hills RK, Lamb K, et al. The importance of relative mutant level for evaluating impact on outcome of KIT, FLT3 and CBL mutations in core-binding factor acute myeloid leukemia. *Leukemia*. 2013;27(9):1891-1901.
5. Andrade FG, Noronha EP, Brisson GD, et al. Molecular characterization of pediatric acute myeloid leukemia: results of a multicentric study in Brazil. *Arch Med Res*. 2016;47(8):656-667.
6. Bang SM, Ahn JY, Park J, et al. Low frequency and variability of FLT3 mutations in Korean patients with acute myeloid leukemia. *J Korean Med Sci*. 2008;23(5):833-837.
7. Bertoli S, Tavitian S, Huynh A, et al. Improved outcome for AML patients over the years 2000-2014. *Blood Cancer J.* 2017;7(12):635.
8. Bezerra MF, Lima AS, Piqué-Borràs MR, et al. Co-occurrence of DNMT3A, NPM1, FLT3 mutations identifies a subset of acute myeloid leukemia with adverse prognosis. *Blood*. 2020;135(11):870-875.
9. Bhatnagar B, Kohlschmidt J, Mrózek K, et al. Poor survival and differential impact of genetic features of Black patients with acute myeloid leukemia. *Cancer Discov*. 2021;11(3):626-637.
10. Bradstock KF, Link E, Di Iulio J, et al. Idarubicin dose escalation during consolidation therapy for adult acute myeloid leukemia. *J Clin Oncol*. 2017;35(15):1678-1685.
11. Byun JM, Kim YJ, Yoon HJ, et al. Cytogenetic profiles of 2806 patients with acute myeloid leukemia-a retrospective multicenter nationwide study. *Ann Hematol*. 2016;95(8):1223-1232.
12. Chou WC, Lei WC, Ko BS, et al. The prognostic impact and stability of Isocitrate dehydrogenase 2 mutation in adult patients with acute myeloid leukemia. *Leukemia*. 2011;25(2):246-253.
13. Daher Reyes GS, Young T, Atenafu E, et al. Risk factors and clinical outcomes of AML patients with central nervous system involvement. *HemaSphere*. 2020;4(S1):265.
14. Damiani D, Tiribelli M, Raspadori D, et al. Clinical impact of CD200 expression in patients with acute myeloid leukemia and correlation with other molecular prognostic factors. *Oncotarget*. 2015;6(30):30212-30221.
15. Damm F, Oberacker T, Thol F, et al. Prognostic importance of histone methyltransferase MLL5 expression in acute myeloid leukemia. *J Clin Oncol*. 2011;29(6):682-689.
16. Damm F, Bunke T, Thol F, et al. Prognostic implications and molecular associations of NADH dehydrogenase subunit 4 (ND4) mutations in acute myeloid leukemia. *Leukemia*. 2012;26(2):289-295.
17. Daver N, Liu Dumlao T, Ravandi F, et al. Effect of NPM1 and FLT3 mutations on the outcomes of elderly patients with acute myeloid leukemia receiving standard chemotherapy. *Clin Lymphoma Myeloma Leuk*. 2013;13(4):435-440.
18. Díaz-Santa J, Rodríguez-Romanos R, Coll R, et al. 5'-nucleotidase, cytosolic II genotype, and clinical outcome in patients with acute myeloid leukemia with intermediate-risk cytogenetics. *Eur J Haematol*. 2022;109(6):755-764.
19. Dickson GJ, Bustraan S, Hills RK, et al. The value of molecular stratification for CEBPA(DM) and NPM1(MUT) FLT3(WT) genotypes in older patients with acute myeloid leukaemia. *Br J Haematol*. 2016;172(4):573-580.
20. DiNardo CD, Ravandi F, Agresta S, et al. Characteristics, clinical outcome, and prognostic significance of IDH mutations in AML. *Am J Hematol*. 2015;90(8):732-736.
21. Döhner K, Schlenk RF, Habdank M, et al. Mutant nucleophosmin (NPM1) predicts favorable prognosis in younger adults with acute myeloid leukemia and normal cytogenetics: interaction with other gene mutations. *Blood*. 2005;106(12):3740-3746.
22. Dufour A, Schneider F, Metzeler KH, et al. Acute myeloid leukemia with biallelic CEBPA gene mutations and normal karyotype represents a distinct genetic entity associated with a favorable clinical outcome. *J Clin Oncol*. 2010;28(4):570-577.
23. Engen C, Hellesøy M, Grob T, et al. FLT3-ITD mutations in acute myeloid leukaemia - molecular characteristics, distribution and numerical variation. *Mol Oncol*. 2021;15(9):2300-2317.
24. Fröhling S, Schlenk RF, Stolze I, et al. CEBPA mutations in younger adults with acute myeloid leukemia and normal cytogenetics: prognostic relevance and analysis of cooperating mutations. *J Clin Oncol*. 2004;22(4):624-633.
25. Gaidzik VI, Schlenk RF, Paschka P, et al. Clinical impact of DNMT3A mutations in younger adult patients with acute myeloid leukemia: results of the AML Study Group (AMLSG). *Blood*. 2013;121(23):4769-4777.
26. Gou H, Zhou J, Ye Y, et al. The prevalence and clinical profiles of FLT3-ITD, FLT3-TKD, NPM1, C-KIT, DNMT3A, and CEBPA mutations in a cohort of patients with de novo acute myeloid leukemia from southwest China. *Tumour Biol.* 2016;37(6):7357-7370.
27. Green CL, Evans CM, Zhao L, et al. The prognostic significance of IDH2 mutations in AML depends on the location of the mutation. *Blood*. 2011;118(2):409-412.
28. Heiblig M, Labussière-Wallet H, Nicolini FE, et al. Prognostic value of genetic alterations in elderly patients with acute myeloid leukemia: a single institution experience. *Cancers*. 2019;11(4):570.
29. Hemmati PG, Vuong LG, Terwey TH, et al. Predictive significance of the European LeukemiaNet classification of genetic aberrations in patients with acute myeloid leukaemia undergoing allogeneic stem cell transplantation. *Eur J Haematol*. 2017;98(2):160-168.
30. Herborg LL, Nederby L, Brøndum RF, Hansen M, Hokland P, Roug AS. Antigen expression varies significantly between molecular subgroups of acute myeloid leukemia patients: clinical applicability is hampered by establishment of relevant cutoffs. *Acta Haematol*. 2021;144(3):275-284.
31. Hidaka D, Onozawa M, Hashiguchi J, et al. Wilms tumor 1 expression at diagnosis correlates with genetic abnormalities and polymorphism but is not independently prognostic in acute myelogenous leukemia: a Hokkaido Leukemia Net study. *Clin Lymphoma Myeloma Leuk*. 2018;18(11):e469-e479.
32. Ho PA, Zeng R, Alonzo TA, et al. Prevalence and prognostic implications of WT1 mutations in pediatric acute myeloid leukemia (AML): a report from the Children's Oncology Group. *Blood*. 2010;116(5):702-710.
33. Ho PA, Alonzo TA, Gerbing RB, et al. The prognostic effect of high diagnostic WT1 gene expression in pediatric AML depends on WT1 SNP rs16754 status: report from the Children's Oncology Group. *Pediatr Blood Cancer*. 2014;61(1):81-88.
34. Hou HA, Chou WC, Kuo YY, et al. TP53 mutations in de novo acute myeloid leukemia patients: longitudinal follow-ups show the mutation is stable during disease evolution. *Blood Cancer J.* 2015;5(7):e331.
35. How J, Sykes J, Gupta V, et al. Influence of FLT3-internal tandem duplication allele burden and white blood cell count on the outcome in patients with intermediate-risk karyotype acute myeloid leukemia. *Cancer*. 2012;118(24):6110-6117.
36. Itzykson R, Duployez N, Fasan A, et al. Clonal interference of signaling mutations worsens prognosis in core-binding factor acute myeloid leukemia. *Blood*. 2018;132(2):187-196.
37. Itzykson R, Fournier E, Berthon C, et al. Genetic identification of patients with AML older than 60 years achieving long-term survival with intensive chemotherapy. *Blood*. 2021;138(7):507-519.
38. Jongen-Lavrencic M, Grob T, Hanekamp D, et al. Molecular minimal residual disease in acute myeloid leukemia. *N Engl J Med*. 2018;378(13):1189-1199.
39. Kandeel EZ, El Sayed G, Elsharkawy N, et al. Impact of FLT3 receptor (CD135) detection by flow cytometry on clinical outcome of adult acute myeloid leukemia patients. *Clin Lymphoma Myeloma Leuk.* 2018;18(8):541-547.
40. Kayser S, Döhner K, Krauter J, et al. The impact of therapy-related acute myeloid leukemia (AML) on outcome in 2853 adult patients with newly diagnosed AML. *Blood*. 2011;117(7):2137-2145.
41. Khanolkar RA, Faridi RM, Kinzel M, et al. Impact of FLT3 internal tandem duplication and NPM1 mutations in acute myeloid leukemia treated with allogeneic hematopoietic cell transplantation. *Cytotherapy*. 2022;24(4):413-420.
42. Koszarska M, Meggyesi N, Bors A, et al. Medium-sized FLT3 internal tandem duplications confer worse prognosis than short and long duplications in a non-elderly acute myeloid leukemia cohort. *Leuk Lymphoma*. 2014;55(7):1510-1517.
43. Kövy P, Őrfi Z, Bors A, et al. Nucleophosmin1 and isocitrate dehydrogenase 1 and 2 as measurable residual disease markers in acute myeloid leukemia. *PLoS One*. 2021;16(6):e0253386.
44. Kurosawa S, Yamaguchi H, Yamaguchi T, et al. Decision analysis of postremission therapy in cytogenetically intermediate-risk acute myeloid leukemia: the impact of flt3 internal tandem duplication, nucleophosmin, and CCAAT/enhancer binding protein alpha. *Biol Blood Marrow Transplant*. 2016;22(6):1125-1132.
45. Li W, Cui L, Gao C, et al. DNMT3A mutations in Chinese childhood acute myeloid leukemia. *Medicine.* 2017;96(31):e7620.
46. Li Y, Shao H, Da Z, Pan J, Fu B. High expression of SLC38A1 predicts poor prognosis in patients with de novo acute myeloid leukemia. *J Cell Physiol*. 2019;234(11):20322-20328.
47. Lima AS, de Mello MR, Fernandes E, et al. Clinical outcomes of patients with acute myeloid leukemia: evaluation of genetic and molecular findings in a real-life setting. *Blood*. 2015;126(15):1863-1865.
48. Linch DC, Hills RK, Burnett AK, Russell N, Gale RE. Analysis of the clinical impact of NPM1 mutant allele burden in a large cohort of younger adult patients with acute myeloid leukaemia. *Br J Haematol*. 2020;188(6):852-859.
49. Löwenberg B, Pabst T, Maertens J, et al. Therapeutic value of clofarabine in younger and middle-aged (18-65 years) adults with newly diagnosed AML. *Blood*. 2017;129(12):1636-1645.
50. Ma QL, Wang JH, Wang YG, et al. High IDH1 expression is associated with a poor prognosis in cytogenetically normal acute myeloid leukemia. *Int J Cancer*. 2015;137(5):1058-1065.
51. Marková J, Michková P, Burčková K, et al. Prognostic impact of DNMT3A mutations in patients with intermediate cytogenetic risk profile acute myeloid leukemia. *Eur J Haematol*. 2012;88(2):128-135.
52. McGregor AK, Moulton D, Bown N, et al. Incidence and outcomes for adults diagnosed with acute myeloid leukemia in the north of England: a real world study. *Leuk Lymphoma*. 2016;57(7):1575-1584.
53. Metzeler KH, Dufour A, Benthaus T, et al. ERG expression is an independent prognostic factor and allows refined risk stratification in cytogenetically normal acute myeloid leukemia: a comprehensive analysis of ERG, MN1, and BAALC transcript levels using oligonucleotide microarrays. *J Clin Oncol*. 2009;27(30):5031-5038.
54. Metzeler KH, Herold T, Rothenberg-Thurley M, et al. Spectrum and prognostic relevance of driver gene mutations in acute myeloid leukemia. *Blood*. 2016;128(5):686-698.
55. Miesner M, Haferlach C, Bacher U, et al. Multilineage dysplasia (MLD) in acute myeloid leukemia (AML) correlates with MDS-related cytogenetic abnormalities and a prior history of MDS or MDS/MPN but has no independent prognostic relevance: a comparison of 408 cases classified as "AML not otherwise specified" (AML-NOS) or "AML with myelodysplasia-related changes" (AML-MRC). *Blood*. 2010;116(15):2742-2751.
56. Nagel G, Weber D, Fromm E, et al. Epidemiological, genetic, and clinical characterization by age of newly diagnosed acute myeloid leukemia based on an academic population-based registry study (AMLSG BiO). *Ann Hematol*. 2017;96(12):1993-2003.
57. Niktoreh N, Walter C, Zimmermann M, et al. Mutated WT1, FLT3-ITD, and NUP98-NSD1 fusion in various combinations define a poor prognostic group in pediatric acute myeloid leukemia. *J Oncol*. 2019;2019:1609128.
58. Nomdedéu J, Hoyos M, Carricondo M, et al. Adverse impact of IDH1 and IDH2 mutations in primary AML: experience of the Spanish CETLAM group. *Leuk Res*. 2012;36(8):990-997.
59. Nomdedéu JF, Hoyos M, Carricondo M, et al. Bone marrow WT1 levels at diagnosis, post-induction and post-intensification in adult de novo AML. *Leukemia*. 2013;27(11):2157-2164.
60. Ok CY, Patel KP, Garcia-Manero G, et al. Mutational profiling of therapy-related myelodysplastic syndromes and acute myeloid leukemia by next generation sequencing, a comparison with de novo diseases. *Leuk Res.* 2015;39(3):348-354.
61. Olesen LH, Nyvold CG, Aggerholm A, Nørgaard JM, Guldberg P, Hokland P. Delineation and molecular characterization of acute myeloid leukemia patients with coduplication of FLT3 and MLL. *Eur J Haematol*. 2005;75(3):185-192.
62. Park BG, Chi HS, Park SJ, et al. Clinical implications of non-A-type NPM1 and FLT3 mutations in patients with normal karyotype acute myeloid leukemia. *Acta Haematol*. 2012;127(2):63-71.
63. Paschka P, Schlenk RF, Gaidzik VI, et al. IDH1 and IDH2 mutations are frequent genetic alterations in acute myeloid leukemia and confer adverse prognosis in cytogenetically normal acute myeloid leukemia with NPM1 mutation without FLT3 internal tandem duplication. *J Clin Oncol*. 2010;28(22):3636-3643.
64. Pastore F, Dufour A, Benthaus T, et al. Combined molecular and clinical prognostic index for relapse and survival in cytogenetically normal acute myeloid leukemia. *J Clin Oncol*. 2014;32(15):1586-1594.
65. Pløen GG, Nederby L, Guldberg P, et al. Persistence of DNMT3A mutations at long-term remission in adult patients with AML. *Br J Haematol*. 2014;167(4):478-486.
66. Renneville A, Abdelali RB, Chevret S, et al. Clinical impact of gene mutations and lesions detected by SNP-array karyotyping in acute myeloid leukemia patients in the context of gemtuzumab ozogamicin treatment: results of the ALFA-0701 trial. *Oncotarget*. 2014;5(4):916-932.
67. Rockova V, Abbas S, Wouters BJ, et al. Risk stratification of intermediate-risk acute myeloid leukemia: integrative analysis of a multitude of gene mutation and gene expression markers. *Blood*. 2011;118(4):1069-1076.
68. Röllig C, Thiede C, Gramatzki M, et al. A novel prognostic model in elderly patients with acute myeloid leukemia: results of 909 patients entered into the prospective AML96 trial. *Blood*. 2010;116(6):971-978.
69. Rubio P, Campos B, Digiorge JA, et al. NPM1, FLT3 and CEBPA mutations in pediatric patients with AML from Argentina: incidence and prognostic value. *Int J Hematol*. 2016;104(5):582-590.
70. Sakaguchi M, Yamaguchi H, Kuboyama M, et al. Significance of FLT3-tyrosine kinase domain mutation as a prognostic factor for acute myeloid leukemia. *Int J Hematol*. 2019;110(5):566-574.
71. Santos FP, Jones D, Qiao W, et al. Prognostic value of FLT3 mutations among different cytogenetic subgroups in acute myeloid leukemia. *Cancer*. 2011;117(10):2145-2155.
72. Sarojam S, Vijay S, Raveendran S, Sreedharan J, Narayanan G, Sreedharan H. FLT3 mutation as a significant prognostic marker in de novo acute myeloid leukemia patients: incidence, distribution and association with cytogenetic findings in a study from South India. *Middle East J Cancer*. 2014;5(4):185-196.
73. Sasaki K, Kanagal-Shamanna R, Montalban-Bravo G, et al. Impact of the variant allele frequency of ASXL1, DNMT3A, JAK2, TET2, TP53, and NPM1 on the outcomes of patients with newly diagnosed acute myeloid leukemia. *Cancer*. 2020;126(4):765-774.
74. Schlenk RF, Döhner K, Kneba M, et al. Gene mutations and response to treatment with all-trans retinoic acid in elderly patients with acute myeloid leukemia. Results from the AMLSG Trial AML HD98B. *Haematologica*. 2009;94(1):54-60.
75. Schnittger S, Schoch C, Kern W, et al. Nucleophosmin gene mutations are predictors of favorable prognosis in acute myelogenous leukemia with a normal karyotype. *Blood*. 2005;106(12):3733-3739.
76. Song IC, Jo DY, Kim HJ, et al. Clinical features and outcomes of hypocellular acute myeloid leukemia in adults: a Korean AML registry data. *Medicine.* 2021;100(1):e24185.
77. Su L, Gao SJ, Li W, et al. NPM1, FLT3-ITD, CEBPA, and c-kit mutations in 312 Chinese patients with de novo acute myeloid leukemia. *Hematology*. 2014;19(6):324-328.
78. Thiede C, Koch S, Creutzig E, et al. Prevalence and prognostic impact of NPM1 mutations in 1485 adult patients with acute myeloid leukemia (AML). *Blood*. 2006;107(10):4011-4020.
79. Tian X, Xu Y, Yin J, et al. TET2 gene mutation is unfavorable prognostic factor in cytogenetically normal acute myeloid leukemia patients with NPM1+ and FLT3-ITD - mutations. *Int J Hematol*. 2014;100(1):96-104.
80. Tien FM, Hou HA, Tsai CH, et al. GATA2 zinc finger 1 mutations are associated with distinct clinico-biological features and outcomes different from GATA2 zinc finger 2 mutations in adult acute myeloid leukemia. *Blood Cancer J*. 2018;8(9):87.
81. Versluis J, In 't Hout FE, Devillier R, et al. Comparative value of post-remission treatment in cytogenetically normal AML subclassified by NPM1 and FLT3-ITD allelic ratio. *Leukemia*. 2017;31(1):26-33.
82. Wan L, Ding S, Xu M, et al. Adverse impact of a high allelic burden FLT3-ITD mutation on allogeneic hematopoietic stem cell transplantation in patients with cytogenetically normal AML. *Int J Hematol*. 2022;116(5):731-743.
83. Wang Xiuli, Haiping Dai, Qian Wang, et al. EZH2 mutations are related to low blast percentage in bone marrow and-7/del (7q) in de novo acute myeloid leukemia. *PLoS One*. 2013;8(4):e61341.
84. Wang X, Chen X, Yang Z, et al. Correlation of TET2 SNP rs2454206 with improved survival in children with acute myeloid leukemia featuring intermediate-risk cytogenetics. *Genes Chromosomes Cancer.* 2018;57(8):379-386.
85. Wang H, Li XQ, Chu TT, et al. Clinical significance of FLT3-ITD/CEBPA mutations and minimal residual disease in cytogenetically normal acute myeloid leukemia after hematopoietic stem cell transplantation. *J Cancer Res Clin Oncol*. 2021;147(9):2659-2670.
86. Wang M, Wang R, Wang H, et al. Difference in gene mutation profile in patients with refractory/relapsed versus newly diagnosed acute myeloid leukemia based on targeted next-generation sequencing. *Leuk Lymphoma*. 2021;62(10):2416-2427.
87. Wattad M, Weber D, Döhner K, et al. Impact of salvage regimens on response and overall survival in acute myeloid leukemia with induction failure. *Leukemia*. 2017;31(6):1306-1313.
88. Wen XM, Lin J, Yang J, et al. Double CEBPA mutations are prognostically favorable in non-M3 acute myeloid leukemia patients with wild-type NPM1 and FLT3-ITD. *Int J Clin Exp Pathol.* 2014;7(10):6832-6840.
89. Xu Y, Sun Y, Shen H, et al. Allogeneic hematopoietic stem cell transplantation could improve survival of cytogenetically normal adult acute myeloid leukemia patients with DNMT3A mutations. *Am J Hematol*. 2015;90(11):992-997.
90. Xu M, Zhao XL, Zhu Y, et al. ND4 mutations are more prevalent in patients with acute myeloid leukemia of M2 morphology. *Transl Cancer Res.* 2018;7(4):1064-1071.
91. Xu LH, Wang JW, Wang Y, Yang FY. Hyperleukocytosis predicts inferior clinical outcome in pediatric acute myeloid leukemia. *Hematology*. 2020;25(1):507-514.
92. Yamato G, Shiba N, Yoshida K, et al. ASXL2 mutations are frequently found in pediatric AML patients with t(8;21)/ RUNX1-RUNX1T1 and associated with a better prognosis. *Genes Chromosomes Cancer*. 2017;56(5):382-393.
93. You E, Cho YU, Jang S, et al. Frequency and clinicopathologic features of RUNX1 mutations in patients with acute myeloid leukemia not otherwise specified. *Am J Clin Pathol.* 2017;148(1):64-72.
94. Yu J, Li Y, Li T, et al. Gene mutational analysis by NGS and its clinical significance in patients with myelodysplastic syndrome and acute myeloid leukemia. *Exp Hematol Oncol*. 2020;9:2.
95. Zaker F, Mohammadzadeh M, Mohammadi M. Detection of KIT and FLT3 mutations in acute myeloid leukemia with different subtypes. *Arch Iran Med*. 2010;13(1):21-25.
96. Zhang Y, Chen Q, Tang G, et al. Allogeneic hematopoietic stem cells transplantation improves the survival of intermediate-risk acute myeloid leukemia patients aged less than 60 years. *Ann Hematol*. 2019;98(4):997-1007.
97. Zhang Y, Wang F, Chen X, et al. Mutation profiling of 16 candidate genes in de novo acute myeloid leukemia patients. *Front Med.* 2019;13(2):229-237.
98. Zhang T, Bao X, Qiu H, et al. Development of a nomogram for predicting the cumulative incidence of disease recurrence of AML after allo-HSCT. *Front Oncol*. 2021;11:732088.
99. Zwaan CM, Meshinchi S, Radich JP, et al. *FLT3* internal tandem duplication in 234 children with acute myeloid leukemia: prognostic significance and relation to cellular drug resistance. *Blood*. 2003;102(7):2387-2394.
